# The Effects of Tai Chi on the Executive Functions and Physical Fitness in Middle-Aged Adults with Depression: A Randomized Controlled Trial

**DOI:** 10.1155/2022/1589106

**Published:** 2022-09-13

**Authors:** Ligong Zhang, Dongshi Wang, Chun Xie, Siwen Liu, Lin Chi, Xuezhi Ma, Fei-Fei Ren

**Affiliations:** ^1^China Wushu School, Beijing Sport University, Beijing, China; ^2^Faculty of Sports Science, Ningbo University, Ningbo, China; ^3^Department of Physical Education, Shanghai Jiao Tong University, Shanghai, China; ^4^China Swimming College, Beijing Sport University, Beijing, China; ^5^School of Physical Education, Minnan Normal University, Zhangzhou, Fujian, China; ^6^Department of Physical Education, Beijing Language and Culture University, Beijing, China

## Abstract

**Objective:**

The present study examined the effects of Tai Chi exercise on the executive functions (EFs) and physical fitness of middle-aged adults with depression.

**Methods:**

A total of 39 middle-aged adults with depression (*M*_age_ = 50.59, *SD* = 7.38) were randomly assigned to the Tai Chi group (*n* = 20) or the waiting-list control group (*n* = 19). The Tai Chi group engaged in two 90 min sessions of Tai Chi exercise per week for 12 weeks; the waiting-list control group was asked to maintain their usual daily routines for 12 weeks. Depression symptoms, EFs (i.e., inhibitory control, planning, working memory, and cognitive flexibility), and physical fitness (i.e., cardiovascular fitness, muscular strength, muscular endurance, power, and flexibility) were evaluated at the baseline (pretest), 6-week (mid-test), and 12-week (post-test) marks.

**Results:**

Both groups showed decreased depression symptoms over time. Compared with the control group, the Tai Chi group showed decreased reaction times for incongruent conditions in the Stroop test from pretest to mid- and post-test, and shorter reaction time for incongruent conditions in the Stroop test than the control group at post-test; the Tai Chi group performed significantly better than the control group in overall total move score of Tower of London (TOL). The Tai Chi group also showed increased total correct scores of TOL from pretest to mid- and post-test, and greater total correct scores of TOL than the control group at post-test. Additionally, results indicated that Tai Chi exercise comprehensively improved physical fitness from pretest to mid- and post-test. Greater performance in terms of cardiovascular fitness, muscular strength, and power was also found in the Tai Chi group at post-test than in the control group.

**Conclusions:**

These findings suggest that the 12-week Tai Chi exercise improved inhibitory control, planning and working memory aspects of executive functions, and physical fitness in middle-aged adults with depression.

## 1. Introduction

Depression is a prevalent and complicated psychiatric disorder [1, 2], mainly characterized by a wide range of symptoms, including but not limited to a persistent low mood or sadness [3], loss of interest in activities [4], hopelessness [5], low self-esteem [6], thoughts of suicide [7], and significant cognitive impairment [8]. It is estimated that over 350 million individuals currently suffer from depression, and the numbers are continuously rising [9]. The lifetime prevalence of depression is 16% of the population in the United States [10] and 6.9% of the population in China [11]. Depression not only results in substantial healthcare costs and financial burdens to patients and society [12], but also causes individuals to suffer tremendously [13] and to have a lower quality of life [14], as well as a shorter life expectancy [15]. Furthermore, the majority of individuals with depression often remain untreated [16] and undiagnosed [17]. In addition, the severity of their symptoms fluctuates over time, putting them at a higher risk of disability and suicide [16, 18]. Depression has become one of the world's most pressing and serious public health concerns [19], and the major cause of disabilities, globally [20].

Previous studies have revealed that depression is associated with cognitive dysfunction and decreased physical fitness [16, 21]. Depressed people frequently show signs of cognitive dysfunction [22]; it is estimated that about two-thirds of people with depression have cognitive dysfunction [23]. Cognitive dysfunction was found to occur 85–94 percent of the time during episodes of depression, and 39–44 percent of the time in remissions for patients with major depressive disorder (MDD) [24]. Depressed people display deficits in multiple cognitive domains, including decreased attention, memory, psychomotor speed, learning, verbal processing, and executive function [25, 26]. A meta-analysis indicated that patients with depression experience significant moderate cognitive dysfunction in executive function, attention, and memory when compared to healthy control subjects [23]. In addition, previous studies have suggested that the executive function deficits of patients with depression were particularly obvious, and those people had significantly worse performance than control groups on the Stroop Color-Word test [27], Trail Making Test-*B* [28, 29], and 2-back test [30]. These tasks are usually used to measure inhibitory control, shifting, and working memory, respectively [30, 31].

Cognitive function is critical in supporting depressed individuals' independence and quality of life. Although antidepressants are the most commonly prescribed treatment for depression [32], existing antidepressant medications have limited or selective efficacy in improving cognitive function in depressed patients [33]. A meta-analysis confirmed that antidepressants only have a small effect on psychomotor speed and delayed recall and do not significantly affect executive function or cognitive control [34]. Additionally, antidepressant medications have poor adherence, low rates of remission, high dropout rates [35], and side effects, which include weight gain [36], nausea, headaches, somnolence [37], increased diabetes risk, and sexual dysfunction [38]. Aside from cognitive dysfunction, studies observed that physical fitness, such as peak oxygen consumption, maximal workload, and individual anaerobic threshold, endurance capacity, and lower and upper limb strength were significantly decreased in depressed patients, compared with healthy controls [39]. Since cognitive dysfunction and decreased physical fitness have a negative impact on the quality of life and functionality of depressed patients [40], finding novel, safe, and effective non-pharmacological interventions to enhance cognitive function and physical fitness for people with depression is of paramount importance.

Tai Chi, as one of the non-pharmacological interventions, is a multimodal and low-intensity Chinese mind-body exercise, which combines cardiovascular and motor fitness, movement coordination, cognitive activity, mindfulness meditation, and social interaction during practice [41, 42]. Tai chi is safe, easy to learn [43], and a popular type of exercise worldwide [44]. It has been shown to be beneficial for cognitive function [45, 46], physical fitness [47, 48], and the symptoms of mood disorders [49] in different populations. Tai Chi has been used as a treatment option for depression by clinicians and scientific researchers, and several randomized controlled trials (RCTs) have indicated that Tai Chi is an effective intervention for reducing depressive symptoms among depressed patients aged 60 years and older [50–52].

Although encouraging results have been observed in older depressed patients, comparatively few studies have been conducted on younger depressed patients. The results from prior studies of Tai Chi for depressive symptoms in patients ranging in age from 18 to 70 years have been inconsistent [53, 54]. More importantly, research on Tai Chi's potential for improving the cognition of depressed patients is still in its infancy. At present, to the best of our knowledge, only a few studies have been carried out on this phenomenon. A randomized controlled study showed that depressed patients aged 60 and above in the Tai Chi plus escitalopram group had significant improvements in memory compared with those receiving health education and escitalopram [51]. Another RCT indicated that no significant improvements in delayed recall, attention/executive function, and language were found for patients aged 60 and older in a Tai Chi group, compared with those in a health education group [50]. Although Tai Chi is a promising intervention for depressive symptoms, most research has been conducted on older patients with depression. The effects of Tai Chi as a means of reducing depressive symptoms, improving cognitive function, and physical health in younger depressed adults have yet to be established.

The purpose of the current randomized controlled study was to investigate whether Tai Chi exercise may substantially improve depressive symptoms, executive functions, and physical fitness in individuals diagnosed with depression. We hypothesized that Tai Chi would improve depressive symptoms, executive functions, and physical fitness in middle-aged adults with depression.

## 2. Materials and Methods

### 2.1. Participants

A total of 39 adults with depression residing in Hong Kong, China, were recruited into this study. Participants' age range was 30–60 years (*M*_age_ = 50.59, *SD* = 7.38). A total of 89.74% were females (*n* = 35). All participants were members of the Dance with Depression Association, a charity organization run by social workers, counselors, psychologists, and physicians that aims to provide free support services to individuals with depression. Participants were recruited through the association's online website and social media with its approval. Potential participants who expressed an interest in this study were invited to complete the Beck Depression Inventory (BDI-II version) and a basic information questionnaire to check whether they met the following eligibility criteria: (1) a BDI-II version score between 14 and 50 points, (2) fulfilled the diagnostic criteria for major depressive disorder (MDD) using the Structured Clinical Interview for the Diagnostic and Statistical Manual of Mental Disorders, Fourth Edition, (3) no history of cardiovascular disease, and (4) normal vision and color perception. All participants signed an informed consent form prior to the intervention. This study was approved by the Institutional Review Board (approval number: C106147). The data collection period was from August 2018 to July 2019. The participants' background data are presented in Table 1.

### 2.2. Procedure

A randomized controlled trial with a multiple time-point experimental design was used in this study. Before the experiments officially began, participants were notified by phone about the experimental procedures and their willingness to participate was confirmed. Those who expressed interest in participating then provided their questionnaire responses to check whether they met the eligibility criteria. The eligible participants were then randomly assigned to a Tai Chi group (*n* = 20, *M*_age_ = 47.20, *SD* = 6.99) and a waiting-list control group (*n* = 19, *M*_age_ = 54.16, *SD* = 6.09). Prior to the intervention, all participants engaged in a series of cognitive and physical fitness tests to assess the effects of the Tai Chi intervention on their executive functions and physical fitness. All participants were said to: (1) have a good, or enough, sleep (at least 7 hours) the night prior to the tests, (2) avoid any physical exercise within 48 hours before the tests, (3) avoid consuming food or beverages containing caffeine, alcohol, and also tea, at least 12 hours prior to attending the tests, and (4) abstain from food for at least one hour prior to the tests.

In the cognitive tests, participants' executive functions, e.g., inhibitory control, working memory, planning skills, and cognitive flexibility, were tested; in the physical fitness tests, participants' cardiovascular fitness, muscular strength, muscular endurance, power, and flexibility were evaluated. After completing the pretest, the participants in the Tai Chi group participated in a three-month Tai Chi training intervention, while those in the control group received no intervention and were asked to maintain their usual daily routines. In addition, all participants completed the BDI-II version six weeks into the intervention and 12 weeks after its conclusion to assess the changes in their depressive state, cognitive test scores, and physical fitness levels. All tests, including the pre-, mid-, and post-tests, took place at the same time of day (9 : 00 AM–16 : 00 PM), and each participant was individually tested in a normally illuminated and quiet room. Those who completed the entire experiment received HKD 600 as a reward.

### 2.3. Intervention Program

The Tai Chi intervention program in this study was designed based on the model developed by Chang et al. [41] in which Tai Chi is thought to be able to change one's cognitive function. The Tai Chi program aims to enhance participants' neurocognitive functions by improving their cardiovascular fitness, motor fitness, movement coordination, social interaction, and meditation status. Thus, a three-month (12-week) Tai Chi program was designed to cater to these five dimensions. During the 90-minute twice-weekly sessions, the participants engaged in 15 minutes of warm-ups (cardiovascular fitness, CRF) that included 3 minutes of jogging and 12 minutes of Tai Chi footwork practice; 10 minutes of Tai Chi kicking and stretching (motor fitness) that included left and right heel kicks, Tai Chi bow stance stretching, Tai Chi horse-riding stance stretching, and Tai Chi crouch stance stretching; 40 minutes of 10-form Tai Chi (movement coordination); 15 minutes of Tai Chi interaction, which included Tai Chi pushing-hands, Tai Chi sharing, Tai Chi grouping practice, and Tai Chi game playing (social interaction); and 10 minutes of Tai Chi meditation (meditation status).

In order to conform to the above model design, the Dong Yue (East Mountain) style of Tai Chi [55], which was created by professors Hui-Feng Men and Gui-Xiang Kan of Beijing Sport University in China, was used in the present study. Ten basic, common Dong Yue Tai Chi movements were selected and modified by an experienced Tai Chi instructor: (1) Qi Shi (Commencing Form); (2) Huai Bao Ri Yue (Embrace the Sun and Moon); (3) Xuan Zhuan Qian Kun-Yi (Reverse the Rotation of the Universe-One); (4) Kai He Zhuang (Arms Open and Close Posture); (5) Xuan Zhuan Qian Kun-Er (Reverse the Rotation of the Universe-Two); (6) Sheng Jiang Zhuang (Rise and Fall Posture); (7) Yun Shou (Cloud Hands); (8) Jin Ji Du Li (Golden Rooster Standing on One Foot); (9) Huai Bao Ri Yue (Embrace the Sun and Moon); and (10) Shou Shi (Closing Form). These movements were selected because they are safe, simple, of low-intensity, and easy to learn.

Beginners could develop their muscle strength, coordination, and balancing skills through the abovementioned activities, which require them to breathe, think, and move simultaneously. Specifically, the Tai Chi program developed in this study entails specialized activities and movements that aim to improve a particular function; for example, jogging and Tai Chi footwork promote participants' cardiovascular fitness, while Tai Chi pushing-hands and group games foster social interactions. The attendance rate of participants in the Tai Chi group over the three-month period (24 sessions in total) was 90.2%.

### 2.4. Measurements

#### 2.4.1. Depression Symptoms

This study adopted the Beck Depression Inventory-II (BDI-II) [56] to assess participants' depression symptoms. The BDI-II is one of the most widely used scales for assessing patients' state of depression and otherwise healthy individuals [57, 58]. It consists of 21 items that measure, on a scale of 0–3 points, the cognitive, affective, somatic, and vegetative symptoms of individuals with depression. An individual's depressive state is assessed based on the total score of all dimensions. The score range is 0–63 points with 0–13 indicating no depression, 14–19 indicating mild depression, 20–28 indicating moderate depression, and 29–63 indicating severe depression. This study adopted the Chinese version of the Beck Depression Inventory version II (BDI-II-C) translated by the Chinese Behavioral Science Corporation (2000). The BDI-II-C has been shown to have good reliability and validity [59].

#### 2.4.2. Executive Functions: Inhibitory Control

This study adopted the paper-pencil version of the Chinese Stroop color-word test as a measure of inhibitory control (Stroop, 1935). Participants are required to respond quickly by identifying different words and colors so as to assess their information processing speed, executive capacity, selective attention, and habitual response inhibition capacity [60]. The Chinese Stroop color-word test in this study consists of two conditions: congruent and incongruent. In the former, the stimuli are color words in which the same color is used to present the meaning of a word; i.e., the word “red” is presented in red, and the word “blue” is presented in blue. In the latter, the stimuli are color words in which a different color is used to present the meaning of a word; i.e., the word “green” is presented in red or blue, while the word “red” is presented in green or blue. In each condition, there are 50 trials shown on an *A*5-size piece of paper. Participants must tell the examiner the color of each trial as quickly and as accurately as they can in a top-to-down and left-to-right pattern. If an error occurs (if the color of a word the participant names differs from the actual color), the examiner informs the participant and requests they repeat the trial until they answer correctly.

The response time in each color-naming condition is taken as an indicator of information processing (congruent condition) and inhibitory control (incongruent condition). The response time is the time taken by a participant to complete all 50 trials. The total test duration is approximately 5 minutes.

#### 2.4.3. Executive Functions: Working Memory and Planning

The Tower of London (TOL) task assesses an individual's working memory and executive planning ability [60]. Participants must move three different-colored wooden balls across three wooden pegs of different lengths to achieve a target position in a minimal number of moves. Specifically, the TOL equipment used in this study is two equally sized wooden boards with three vertical wooden pegs attached and two sets of wooden balls colored red, blue, and green. Prior to starting the test, the examiner arranges each set of balls in the start position on the one board and the target position on the other board. The participant then attempts to use the least number of moves possible to move the balls from the start position to the target position while following three rules: (1) only one ball can be moved at any given time; (2) a ball must be placed on a wooden peg at all times; and (3) the longest peg can accommodate three balls, the middle peg can accommodate two balls, and the shortest peg can only accommodate one ball. The 10-item Tower of London-Drexel University test [61] was used in this study, and the test duration was 15–20 minutes.

Five TOL task performance indicators were adopted in this study: the total move score, total correct score, total initiation time, total execution time, and total problem-solving time [62]. The total move score is the actual number of moves made by a participant minus the minimum possible number of moves. The summation of the score differences of all items was also recorded. The total correct score, which ranges from 0 to 10 points, is the total number of items solved using the minimum number of moves. Since the minimum possible number of moves differs for all items, a participant scores only one point for each item completed using the minimum number of moves. The total initiation time represents the time when a participant began their first move. The total execution time is the time taken by a participant to move their first ball and solve the item. Finally, the total problem-solving time is the sum of the total initiation time and the total execution time. All time-related measurements were calculated in seconds.

#### 2.4.4. Executive Functions: Cognitive Flexibility

This study used the computer version of the Wisconsin Card Sorting Test (WCST) as a measure of cognitive flexibility and other related executive functions [31]. The test consists of four key cards and 128 response cards. At the start of the test, four key cards with different patterns are displayed on the top of the screen in the following order from left to right: one red triangle, two green stars, three yellow crosses, and four blue circles, while the response cards are displayed at the bottom of the screen. During the WCST, participants are instructed to select one response card to match the color, shape, or number of items shown in the key cards by pressing the left and right and up and down arrow keys. Once a pair is formed, the computer will generate a cue tone and display the word “Correct” or “Incorrect” in the lower left corner of the screen. Once a participant completes ten consecutive correct matches, the computerized protocol informs the participant of new classification rules by generating a message in the lower left corner of the screen that displays the new order, i.e., color, shape, and number of items. The test finishes when a participant successfully completes six classifications or all 128 matches. All participants completed the WCST within 30 minutes. The number of categories completed, perseverative response, conceptual-level responses, and failure to maintain set were used as indicators of cognitive flexibility performance in the WCST.

#### 2.4.5. Physical Fitness: Cardiovascular Fitness

In this study, the 3-minute step test was adopted as a measure of CRF [63]. Participants were required to step on and off a 35 cm high box while maintaining a rate of 24 steps per minute (or a cadence of 96 beats per minute) for three minutes until the conclusion of the test; their heart rates were then measured at 1–1.5 minutes, 2–2.5 minutes, and 3–3.5 minutes. When a participant loses his or her rhythm more than three times or is unable to continue stepping onto the box, the test immediately ends. To ensure safety, the examiner instructs each participant about the correct stepping procedure (such as keeping the upper body straight when stepping on, keeping both feet on the surface of the box, and refraining from jumping). The indicator of the step test is calculated through the following formula: (the total time of the step test (in seconds) × 100)/(2 × (sum total of three measured heart rates)). A higher test score indicated better cardiorespiratory fitness.

#### 2.4.6. Physical Fitness: Muscular Strength and Muscular Endurance

Participants' muscular strength and muscular endurance were measured through the curl-up test. During the test, participants had to complete as many curl-ups as possible within one minute. They were instructed to lie flat on a soft pad with the knees bent at 90 degrees, arms crossed over the chest, and palms resting on both shoulders. The examiner then pressed their hands on the participant's instep to provide additional stability. The number of curl-ups completed was taken as the number of times a participant moves their upper body upwards by contracting the abdominal muscles, touches both knees with the elbows, and returns to the starting supine position by relaxing the abdominal muscles. The number of curl-ups performed in the first and last 30 seconds of the test was taken as an indicator of muscle strength and muscle endurance, respectively.

#### 2.4.7. Physical Fitness: Power

In this study, participants' lower limb power was measured through the standing long jump test. Participants were instructed to stand at the starting line with feet shoulder-width apart. Before jumping, they had to half-squat, bend their knee joints, and position their arms laterally behind their backs. When jumping, they had to swing their arms to leap forward and land with both feet reaching the ground at the same time. Participants performed two jumps during the test, and the best result was taken as the indicator of power.

#### 2.4.8. Physical Fitness: Flexibility

The sit-and-reach test in this study evaluates the flexibility of the participants' hamstrings and lumbar regions. During the test, participants were instructed to sit on a flat mat and straighten their legs with the toes facing upwards and both heels placed on an arrow-shaped forward stretch indicator. They were then instructed to place one palm on top of the other and bend their body forward to push the stretch indicator on the instrument for three seconds, and the distance displayed on the instrument was recorded. Each participant performed this maneuver twice during the test, with the best distance adopted as the flexibility indicator.

### 2.5. Statistical Analysis

This study applied independent-sample *t*-tests and chi-squared tests to compare the demographic data of the two groups. Furthermore, 2 (Group) × 3 (Time: pretest vs. mid-test vs. post-test) repeated-measures analysis of variance (RMANOVA) was employed to examine participants' pretest, mid-test, and post-test differences in terms of depression symptoms, executive function (Stroop color-naming test, TOL, and WCST), and physical fitness (cardiovascular fitness, muscle strength, endurance, power, and flexibility). The level of statistical significance (*α*) was set at 0.05. Post hoc comparisons were performed on significant main effects. Simple main effect comparisons were performed on significant interactions, while multiple comparisons were counteracted by the Bonferroni correction. All statistical analyses were performed using the SPSS version 21.0 statistical software (IBM Corporation, Armonk, NY, USA). All variables were presented in terms of the mean and standard deviation (SD).

## 3. Results

### 3.1. Participant Characteristics

The independent-sample *t*-test results indicated a significant difference between the Tai Chi and the control groups in age (*t* (1, 37) = −0.68, *p* < 0.01). Additionally, the chi-squared test results indicated a significant difference in terms of antidepressant usage between the Tai Chi and control groups (*χ*^2^ (1) = 4.73, *p*=0.03). No significant differences were found between the two groups in terms of other background variables such as gender, height, weight, BMI, education level, duration of depression, and the percentage of patients undergoing psychotherapy (*ps* > 0.05).

### 3.2. BDI-II-C

**Figure 1:**
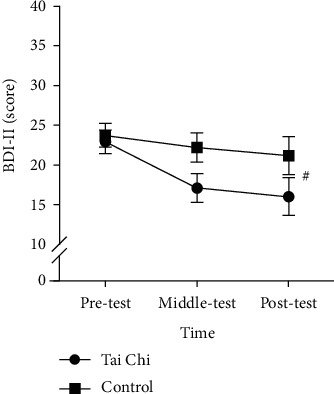
Differences between the Tai Chi group and the control group on the BDI-II score across three time points (mean ± standard error); ^#^represents a significant difference between time points.

The RMANOVA results indicated that time had a significant main effect on the BDI-II-C score [*F* (2, 74) = 10.13, *p* < 0.001, partial eta-squared = 0.22]. Post hoc comparison tests showed that the pretest score (23.32 ± 1.06) was significantly higher than the mid- and post-test scores (19.66 ± 1.31 and 18.55 ± 1.68, *ps* < 0.05), but no significant difference was found between the mid-test and the post-test scores (*p* > 0.05). Furthermore, no significant group differences [*F* (1, 37) = 2.35, *p* > 0.05] and interactions [*F* (2, 74) = 2.54, *p* > 0.05] were observed (see Figure 1).

### 3.3. Stroop Test: Congruent Condition

**Figure 2:**
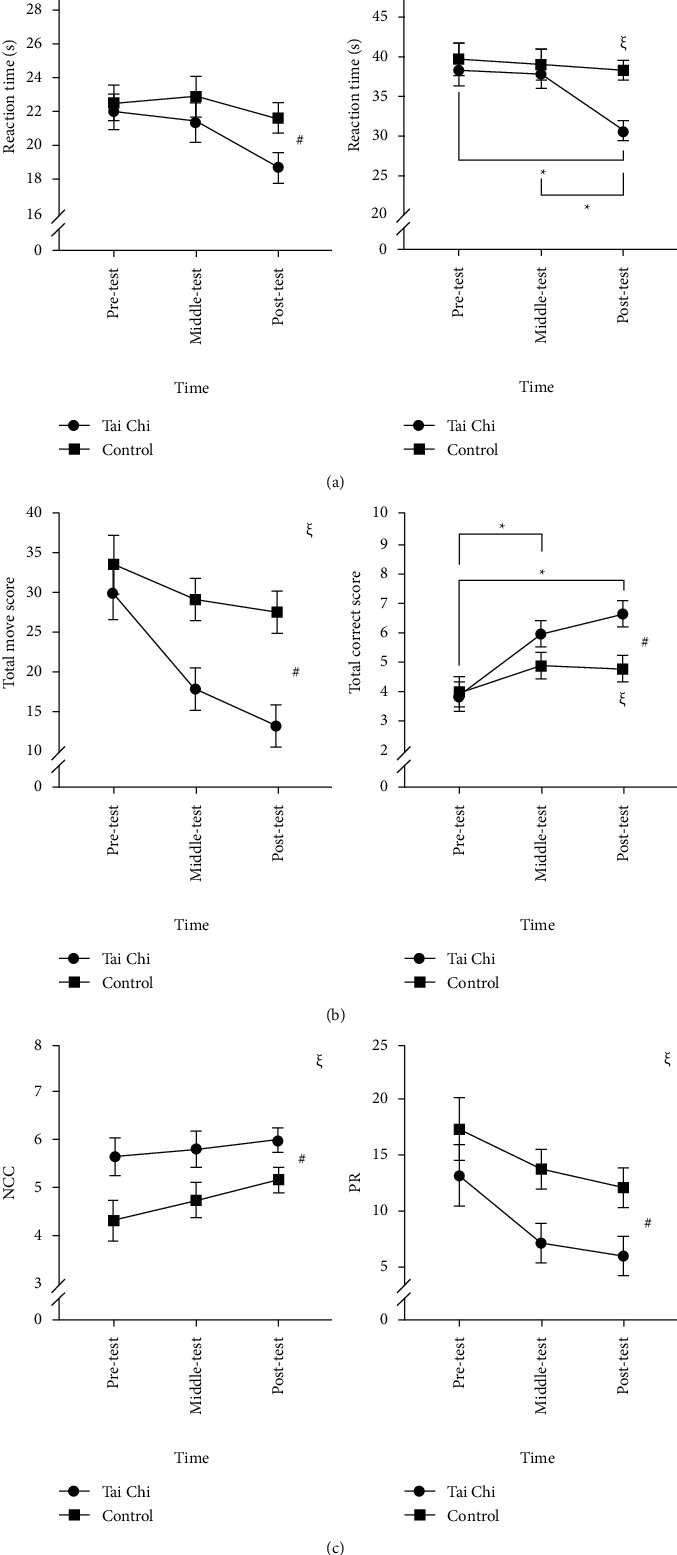
Differences between the Tai Chi group and the control group in (a) reaction times of congruent condition and incongruent condition in the Stroop test, (b) move scores and correct scores of Tower of London (TOL), and (c) number of categories completed (NCC) and perseverative responses (PR) of the Wisconsin Card Sorting Test (WCST) across time points (mean and standard error). ^#^represents a significant difference between time points. ^*∗*^represents a significant difference across time points within the Tai Chi group. ^§^represents a significant difference between groups.

The RMANOVA results indicated that time had a significant main effect on the response time in the congruent condition [*F* (2, 74) = 9.31, *p* < 0.001, partial eta-squared = 0.20]. Post hoc comparison tests showed that the post-test response time (20.18 ± 4.18 s) was significantly faster than the pre- and mid-test times (22.15 ± 5.17 s and 22.33 ± 4.47 s, *ps* < 0.05), but no significant difference was found between the pretest and the mid-test times (*p* > 0.05). No significant group differences [*F* (1, 37) = 2.35, *p* > 0.05] and interactions [*F* (2, 74) = 2.54, *p* > 0.05] were observed (see Figure 2(a)).

### 3.4. Stroop Test: Incongruent Condition

The RMANOVA results indicated the presence of a significant group-time interaction in terms of the response time in the incongruent condition [*F* (2, 74) = 6.15, *p* < 0.05, partial eta-squared = 0.14]. Post hoc comparison tests showed that the Tai Chi group's post-test response time in the incongruent condition (30.65 ± 5.81 s) was significantly faster than that of the control group (38.21 ± 5.09, *p* < 0.001), but no significant differences were found between the pre- and mid-test times in both groups (*p* > 0.05). Additionally, the Tai Chi group's post-test response time in the incongruent condition was significantly faster than their pre- and mid-test response times (38.35 ± 10.47 s and 37.75 ± 9.47 s, *ps* < 0.001), while no significant differences across these three time points were found in the control group (*p* > 0.05). Meanwhile, time had a significant main effect on the response time in the incongruent condition [*F* (2, 74) = 11.25, *p* < 0.001, partial eta-squared = 0.23]. Post hoc comparison tests showed that the post-test response time (34.33 ± 6.62 s) was significantly faster than the pre- and mid-test times (38.97 ± 8.51 s and 38.33 ± 8.30 s, *ps* < 0.05). No significant group main effects were observed [*F* (1, 37) = 2.48, *p* > 0.05] (see Figure 2(a)).

### 3.5. Tower of London Task: Total Move Score

The RMANOVA results indicated that time had a significant main effect on the total move score [*F* (2, 74) = 2.28, *p* < 0.001, partial eta-squared = 0.22]. Post hoc comparison tests showed that the average pretest move score (31.67 ± 16.17) was significantly higher than the mid- and post-test scores (23.26 ± 13.74 and 20.10 ± 13.86, *ps* < 0.05), but no significant difference was found between the mid- and the post-test scores (*p* > 0.05). Furthermore, significant group main effects [*F* (2, 37) = 9.01, *p* < 0.05, partial eta-squared = 0.20] were observed. The average move score of the Tai Chi group (20.23 ± 11.70) was significantly lower than that of the control group (30.04 ± 15.15, *p* < 0.05). However, no significant time-group interactions were observed [*F* (2, 74) = 2.28, *p* > 0.05] (see Figure 2(b)).

### 3.6. Tower of London Task: Total Correct Score

The RMANOVA results indicated that time had a significant main effect on the total correct score [*F* (2, 74) = 18.80, *p* < 0.001, partial eta-squared = 0.34]. Post hoc comparison tests showed that the average pretest move score (3.92 ± 2.17) was significantly lower than the mid-test score (5.44 ± 2.09, *p* < 0.05) and the post-test score (5.74 ± 2.14, *p* < 0.001), but no significant difference was found between the mid- and post-test scores (*p* > 0.05). Furthermore, significant time-group interactions were observed [*F* (2, 74) = 5.20, *p* < 0.05, partial eta-squared = 0.12]. The post-test correct move score of the Tai Chi group (6.65 ± 2.01) was significantly higher than that of the control group (4.79 ± 1.87, *p* < 0.05), but no significant differences were observed in the pre- and mid-test scores of both groups (*ps* > 0.05). The pretest correct move score of the Tai Chi group (3.85 ± 2.25) was significantly lower than the mid-test score (5.95 ± 2.09, *p* < 0.05) and the post-test score (6.65 ± 2.01, *p* < 0.001), but no significant difference was found between the mid- and the post-test scores (*p* > 0.05). In addition, no significant differences in the total correct score across these three time points were found in the control group (*p* > 0.05) (see Figure 2(b)). No significant main effects and interaction effects in the total initiation time, total execution time, and total problem-solving time were observed (*ps* > 0.05).

### 3.7. Wisconsin Card Sorting Test: Number of Categories Completed

The RMANOVA results indicated that time had a significant main effect on the number of categories completed [*F* (2, 74) = 4.63, *p* < 0.05, partial eta-squared = 0.11]. Post hoc comparison tests showed that the number of completions of the post-test (5.58 ± 1.19) was significantly higher than that of the pretest (5.00 ± 1.88, *p* < 0.05), while no significant differences were observed between the number of completions of the mid-test and that of the pretest and post-test (*ps* > 0.05). Furthermore, significant main group effects were observed [*F* (2, 37) = 6.04, *p* < 0.05, partial eta-squared = 0.14], with the average number of categories completed by the Tai Chi group (5.82 ± 0.31) being significantly higher than that of the control group (4.74 ± 0.32, *p* < 0.05). However, no significant time-group interactions were observed [*F* (2, 74) = 5.92, *p* > 0.05] (see Figure 2(c)).

### 3.8. Wisconsin Card Sorting Test: Perseverative Response

The RMANOVA results indicated that time had a significant main effect on perseverative response [*F* (2, 74) = 8.98, *p* < 0.001, partial eta-squared = 0.20]. Post hoc comparison tests showed that the pretest response (15.36 ± 12.54) was significantly higher than the mid- and post-test responses (10.49 ± 8.73 and 9.05 ± 8.55, *ps* < 0.05), but no significant difference was found between the mid- and the post-test responses (*p* > 0.05). Furthermore, significant main group effects were observed [*F* (2, 37) = 4.98, *p* < 0.05, partial eta-squared = 0.12]. The perseverative response of the Tai Chi group (8.83 ± 1.80) was significantly lower than that of the control group (14.58 ± 1.84, *p* < 0.05), but no significant time-group interactions were observed [*F* (2, 74) = 0.37, *p* > 0.05] (see Figure 2(c)). There were no significant main and interaction effects in the conceptual-level responses and a failure to maintain set (*ps* > 0.05).

### 3.9. 3-Minute Step Test

**Figure 3:**
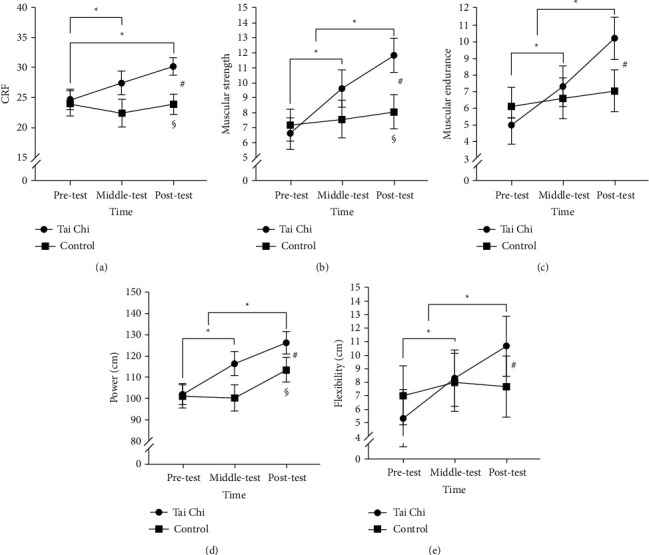
Differences between the Tai Chi group and the control group in physical fitness across time points (mean and standard error); CRF = cardiovascular fitness. ^#^represents a significant difference between time points. ^§^represents a significant difference between groups. ^*∗*^represents a significant difference across time points within the Tai Chi group.

The RMANOVA results indicated that time had a significant main effect on cardiovascular fitness [*F* (2, 74) = 6.53, *p* < 0.05, partial eta-squared = 0.17]. Post hoc comparison tests showed that the post-test score (27.48 ± 7.02) was significantly higher than the pretest score (24.34 ± 7.33, *p* < 0.05). However, no significant differences were observed between the mid-test score (25.28 ± 8.86) and the pre- and post-test scores (*ps* > 0.05). Furthermore, significant time-group interactions were observed [*F* (2, 74) = 6.87, *p* < 0.05, partial eta-squared = 0.18]. The post-test cardiovascular fitness of the Tai Chi group (30.15 ± 3.13) was significantly higher than that of the control group (23.86 ± 9.12, *p* < 0.05), but no significant differences were observed in the pre- and mid-test scores of both groups (*ps* > 0.05). Furthermore, the pretest cardiovascular fitness of the Tai Chi group (24.65 ± 6.60) was significantly lower than the mid-test (27.43 ± 6.61, *p* < 0.05) and post-test (*p* < 0.001) cardiovascular fitness, but no significant difference was found between the mid- and post-test scores (*ps* > 0.05). Further, no significant differences in the total correct score across these three time points were found in the control group (*p* > 0.05) (see Figure 3(a)).

### 3.10. Curl-Up Test

The RMANOVA results indicated that time had a significant main effect on muscle strength [*F* (2, 74) = 20.04, *p* < 0.001, partial eta-squared = 0.37]. Post hoc comparison tests showed that the post-test score (9.94 ± 5.14) was significantly higher than the pretest (6.89 ± 4.46, *p* < 0.001) and mid-test scores (8.58 ± 5.37, *p* < 0.05), while the mid-test score was significantly higher than the pretest score (*p* < 0.05). Furthermore, significant group-time interactions were observed [*F* (2, 74) = 10.18, *p* < 0.001, partial eta-squared = 0.23]. The post-test muscle strength of the Tai Chi group (11.83 ± 4.30) was significantly higher than that of the control group (8.06 ± 5.33, *p* < 0.05), but no significant differences were observed in the pre- and mid-test scores of both groups (*p* > 0.05). Furthermore, the post-test muscle strength of the Tai Chi group was significantly higher than the pretest (6.61 ± 4.53, *p* < 0.001)) and mid-test (9.61 ± 4.72, *p* < 0.05) muscle strength, and the mid-test was significantly higher than the pretest (*p* < 0.001). On the contrary, no significant differences in the muscle strength across these three time points were found in the control group (*p* > 0.05) (see Figure 3(b)).

The results revealed that the mid- and post-test muscle endurance of the Tai Chi group was significantly higher (7.33 ± 4.98 and 10.22 ± 5.57, respectively, *ps* < 0.001) than the pretest muscle endurance (5.00 ± 4.60), with the post-test being significantly higher than the mid-test (*p* < 0.05). On the contrary, no significant differences in the muscle endurance across these three time points were found in the control group (*ps* > 0.05), as well as between both groups (*ps* > 0.05) (see Figure 3(c)).

### 3.11. Standing Long Jump

The RMANOVA results indicated that time had a significant main effect on power [*F* (2, 74) = 16.78, *p* < 0.001, partial eta-squared = 0.34]. Post hoc comparison tests showed that the mid-test (109.12 ± 25.51) and post-test scores (113.56 ± 26.86) were significantly higher than the pretest score (101.65 ± 21.11, *ps* < 0.001), while no significant differences were observed between the mid- and post-test scores (*p* > 0.05). Furthermore, significant group-time interactions were observed [*F* (2, 74) = 25.08, *p* < 0.001, partial eta-squared = 0.43], in which the post-test power of the Tai Chi group (126.21 ± 17.11 cm) was significantly higher than that of the control group (113.56 ± 26.86 cm, *p* < 0.05). However, no significant differences were observed in the pre- and mid-test scores of both groups (*ps* > 0.05). Additionally, the post-test power of the Tai Chi group was significantly higher than the pretest (102.16 ± 17.32 cm, *p* < 0.001) and mid-test (116.51 ± 18.70 cm, *p* < 0.05) power and the mid-test was significantly higher than the pretest (*p* < 0.001). On the contrary, no significant differences in the power across these three time points were found in the control group (*ps* > 0.05), as well as between both groups (*ps* > 0.05) (see Figure 3(d)).

### 3.12. Sit and Reach

The RMANOVA results indicated that time had a significant main effect on flexibility [*F* (2, 74) = 13.09, *p* < 0.001, partial eta-squared = 0.26]. Post hoc comparison tests showed that the mid-test (8.14 ± 9.20) and post-test scores (9.21 ± 9.88) were significantly higher than the pretest score (6.15 ± 9.42, *ps* < 0.05), while no significant differences were observed between the mid- and post-test scores (*p* > 0.05). Furthermore, significant group-time interactions were observed [*F* (2, 74) = 7.69, *p* < 0.05, partial eta-squared = 0.17], with post-test flexibility (10.66 ± 7.84 cm) of the Tai Chi group being significantly higher than the pretest (5.34 ± 7.61 cm, *p* < 0.001) and mid-test flexibility (8.29 ± 8.22 cm, *p* < 0.05) with higher flexibility levels recorded on the mid-test than on the pretest (*p* < 0.05). No significant changes in flexibility were observed in the control group (*ps* > 0.05). Additionally, no significant differences in flexibility across these three time points were found in the control group (*ps* > 0.05) (see Figure 3(e)).

## 4. Discussion

This study examined the effects of a three-month Tai Chi program on the executive functions and physical fitness of depressed adults. The results of the study provide preliminary validation of the effectiveness of the Tai Chi interventional program in improving the executive functions and physical fitness of the depressed adults. Both groups of participants significantly mitigated their depressive symptoms, but only the Tai Chi group improved inhibitory control, working memory, and planning, along with cardiovascular fitness, muscle strength, muscle endurance, power, and flexibility.

Even though no significant benefits of symptom mitigation were conferred to the participants in the Tai Chi group, the results showed that the depression symptoms of the participants in both groups decreased over time. One reason for this may be the higher percentage of antidepressant usage in the control group (see Tables 1 and 2). Previous studies have indicated that antidepressants are effective in mitigating depression symptoms [64]. Our findings are similar to those of a previous study [50], which found that a combination of Tai Chi and health education and drug therapy could alleviate symptoms of geriatric depression. Interestingly, compared with the potential side effects of antidepressants [35, 37], the Tai Chi program was relatively safer and had no adverse effects on depression symptom mitigation [35, 65].

The results of better inhibitory control and planning in the Tai Chi group imply that even though both groups mitigated their depression symptoms, the use of antidepressants did not confer any significant effects on cognitive control and executive function, which is possibly due to different neuroprocessing mechanisms, as suggested in previous studies [34]. Nonetheless, Tai Chi is a multimodal exercise that helps improve various cognitive dimensions, including executive function [66], and may be more effective than other typical forms of exercise (aerobics and resistance training) [67]. These differences suggest that the therapeutic pathways by which Tai Chi improves the executive function of adults with depression confer a wider range of benefits. Tai Chi is a kind of light- or light-to-moderate intensity aerobic exercise, though a large number of studies have shown that regular aerobic exercise can through the movement of the muscle release a lot of muscle factor (such as brain-derived neurotrophic factor) and metabolic product (such as lactate), via the blood to the brain, and the influence of neuron- and glial cell-related functions. This alters neurotransmission in relevant brain regions, such as the frontal and temporal lobes, and enhances executive function [68].

The results of shortened incongruent post-test Stroop color-naming test response time in Tai Chi are in line with those of previous studies on the effectiveness of Tai Chi on elderly individuals [69], methamphetamine dependents [70], and individuals with mild cognitive impairment [71]. However, our study expanded to patients with depression, to examine whether the three-month Tai Chi intervention effectively improved the inhibitory control of adults with depression. Additionally, previous studies have shown that typical forms of long-term exercise such as aerobics [72] and resistance training [73] exercises have positive effects on inhibitory control. In reality, Tai Chi is a multimodal exercise consisting of multiple components that improve cognitive function (e.g., aerobic exercise, muscle exercise, coordination exercise, social interaction, and meditation) [41], and thus may confer additional benefits toward inhibitory control. A study found that multimodal and progressive resistance exercises can improve the inhibitory control of otherwise healthy elderly adults. However, multimodal exercises seemingly affect inhibitory control directly, while progressive resistance exercises affect inhibitory control through muscle strength. This indicates that as opposed to other forms of exercise, Tai Chi and other multimodal exercises promote inhibitory control through various underlying mechanisms. Furthermore, Shen et al. [74] pointed out that compared with aerobic exercise (e.g., speed walking), Tai Chi is more effective for promoting the inhibitory control of elderly individuals and enhanced inhibitory control is associated with an increased fractional amplitude of low-frequency fluctuation (fALFF) of the left medial superior frontal gyrus. Another study that applied functional near-infrared spectroscopy (fNIRS) found that in addition to significantly enhancing inhibitory control, Tai Chi also increases the oxygenated hemoglobin (oxy-Hb) levels in the prefrontal cortex when performing the flanker test. These findings demonstrate that compared with typical forms of exercise, Tai Chi may potentially benefit inhibitory control-related variables such as the activation and processing efficiency of the cranial nerves.

Individuals with depression have shown lower TOL performance [8]. This study found that the total TOL move score of the Tai Chi group was significantly lower than that of the control group, but the total correct score of the Tai Chi group had improved significantly six weeks into the intervention, and the post-test total correct score of the Tai Chi group was significantly higher than that of the control group. These findings suggest that the Tai Chi intervention was conducive to improving executive functions such as planning and working memory in individuals with depression [75, 76]. In general, the Tai Chi group performed fewer moves but improved by a larger margin, a finding that is similar to the results of studies on elderly individuals with cognitive impairment and obese adolescents [75, 77]. This demonstrates that Tai Chi and other multimodal exercises could potentially improve the quality and efficiency of an individual's executive planning skills. Nonetheless, these results should be interpreted with caution because this study did not find any evidence of the Tai Chi intervention improving the group's total initiation time, total execution time, and total problem-solving time. These three indicators represent, respectively, the planning of strategies for determining problem-solving solutions, the quality of executive planning, and the overall speed of executive planning [61, 62]. This implies that the Tai Chi intervention had limited effects on executive planning. Sungkarat et al. [78] found that even though the Tai Chi intervention benefits general memory and conversion, it does not improve planning-related outcomes. Therefore, future studies should explore the effects of Tai Chi on planning-related executive functions to compensate for the existing shortcomings in the research [46, 67].

Furthermore, consistent with the findings of previous meta-analyses on the effects of Tai Chi on executive function [46, 67] a higher post-test total correct score signifies that the Tai Chi intervention can improve working memory. Even though previous empirical results support the effectiveness of long-term aerobic exercise on working memory [79], a recent meta-analysis of 25 RCT studies showed that Tai Chi-centered mind-body exercises can improve working memory more efficiently [72]. The data in general attest to the consistency of findings on the positive effects of Tai Chi on working memory. This study further extends the results to adults with depression, where cerebral cortex damage may be associated with poorer TOL performance (less activation in the bilateral dorsal-lateral prefrontal cortex, the frontal lobe cortex on the rear and external sides when in activity) [8]. Meanwhile, since working memory is associated with maintaining a good quality of life, Tai Chi can be seen as an important measure for improving the working memory of individuals with depression. A cross-sectional study demonstrated that regular Tai Chi training is linked to effective brain functional connections [80]. More specifically, experienced Tai Chi practitioners have more connections in their prefrontal, motor, and occipital cortices than those without experience. Additionally, compared with those doing regular walking exercise, the regular Tai Chi practitioners had higher gray matter density in their temporal regions (i.e., left hippocampus and the adjacent left parahippocampal gyrus), and the Tai Chi practitioners also exhibited higher spontaneous ReHo activation in the left hippocampus and the parahippocampal gyrus [81]. These results implicate that Tai Chi practice might be used to generate neural connectivity patterns and modify the hippocampus's structure and functions that could significantly improve the working memory of individuals with depression.

Even though previous studies have found that regular Tai Chi training can improve the cognitive flexibility of elderly individuals [82], regardless of the number of categories completed or perseverative response and other behavioral indicators, no evidence on the effects and efficiency of Tai Chi in enhancing the WCST performance was found in this study. We suggest that this could be due to the session time, which was set at 90 minutes per session in this study. Previous research has highlighted that Tai Chi sessions longer than 60 minutes may have fewer desirable effects on executive function [46]. For example, some studies found that 50-minute Tai Chi sessions can effectively improve the cognitive flexibility-related performance of elderly individuals with cognitive damage, as well as otherwise healthy adults. Accordingly, overly long training sessions may decrease the benefits of Tai Chi on cognitive flexibility. Alternatively, the selective promoting effects of Tai Chi on executive function may be limited to specific executive functions (e.g., inhibitory control and working memory) that do not include cognitive flexibility. Even though this may be associated with the effects of the type of cognitive tasks as a mediator between physical activity and executive function [83], a recent study found that a 40-minute session Tai Chi intervention had no cognitive flexibility-promoting effects on methamphetamine dependents [70]. The authors of that study suggested that this finding could be due to the short length of the course (12 weeks in total). Future studies should further validate how various lengths and frequencies of Tai Chi interventions affect cognitive flexibility.

In addition, like their depression symptoms, this study found that both groups significantly improved their cognitive flexibility over time. We suggest that this could be associated with the efficacy of antidepressants. That is to say, the negative association between depression symptoms and cognitive flexibility [84] may confound the effectiveness of the Tai Chi intervention on cognitive flexibility. However, controlling antidepressant usage in a clinical study may violate research ethics, as well as the patients' physical and mental health. Therefore, future studies could set antidepressant use as a covariate to eliminate the effects of drug therapy.

In line with previous studies on otherwise healthy adults [85], elderly individuals [86], and methamphetamine-dependent women [70], this study demonstrated that the Tai Chi intervention comprehensively improved the physical fitness of adults with depression. Thus, Tai Chi was found to be conducive to improving an individual's cardiovascular fitness, muscle strength, muscle endurance, power, and flexibility. Based on the Chang et al. [41] model on the association between Tai Chi and cognitive function, improvements in cardiovascular fitness, motor fitness, movement coordination, social interaction, and meditation state may mediate the association between Tai Chi and cerebral function. The results of the study indirectly supported the aforementioned model in which improvements in various physical fitness performances are concomitant with enhanced executive functions such as inhibitory control and working memory. Additionally, during the process of Tai Chi training, participants had to perform various coordination-related movements that involve mindful awareness and the group training may have also affected their social interactions. These cognitive factors could contribute to participants' improved brain function through Tai Chi [41].

Furthermore, enhanced physical fitness may indirectly improve the quality of life of an individual with depression [87]. Some studies found that multimodal exercises [88] and mind-body exercises [89] not only improve multiple physical fitness indicators but also elevate an individual's perceived quality of life. These findings imply that Tai Chi and other related physical exercises are associated with both an individual's executive function and their quality of life.

This study has several limitations. First, even though the participants recruited into this study were confirmed patients with depression as diagnosed by a physician, their baseline BDI-II scores ranged from 14 to 39, which indicates that their depression symptoms include mild-to-severe depression. A previous meta-analysis reported that the severity of depression influences the therapeutic efficacy of physical exercise on depression [90]. Second, even though the participants in the study were grouped randomly, differences in age and antidepressant use between the Tai Chi group and the control group were observed. This may confound the benefits of the Tai Chi intervention on the participants' depression symptoms and executive function [90, 91]. Third, due to the imbalanced gender ratio of the participants, the results of this study should be interpreted cautiously as the lower number of male participants could mean that the results can only be generalized to females with depression. Therefore, future studies on Tai Chi as a means to improve the executive function and physical fitness of individuals with depression should focus on the potential mediation effects of gender. Fourth, this study found that Tai Chi benefits executive functions such as inhibitory control, working memory, and planning. However, similar findings were not observed with regard to cognitive flexibility, which could be attributed to the overly long session time (i.e., longer than 60 min) [46]. Considering the fact that Tai Chi is a multimodal exercise, it is necessary to include multiple components (e.g., Tai Chi stretching, Tai Chi pushing-hands, and Tai Chi meditation) in Tai Chi sessions, but these inclusions will lengthen the session time. In this regard, more research is warranted to validate the dose-response relationship between the length of a Tai Chi session and cognitive flexibility [92]. Lastly, since participants did 3 minutes of jogging during warm-ups in each class, we cannot completely rule out the intervention-induced benefits, and to some extent, the improvement in CRF may also have been derived from jogging. This factor, however, needs to be considered and controlled in future RCT studies.

## 5. Conclusions

This is the first study to explore the effects of Tai Chi on the executive function and physical fitness of adults with depression. The results of the study provide a preliminary validation of the effectiveness of Tai Chi for improving executive functions (e.g., inhibitory control, working memory, and planning) and physical fitness (e.g., cardiovascular fitness, muscle strength, muscle endurance, power, and flexibility) of adults with depression. Moreover, the improvements in a wide range of physical fitness indicators imply that Tai Chi potentially generates executive function-enhancing mechanisms. Future studies should further validate the causal relationships between Tai Chi-improved executive function and physical fitness while also taking into account the impacts of confounding variables so as to provide robust evidence on the effectiveness of Tai Chi as an intervention for depression.

## Figures and Tables

**Table 1:** Demographic characteristics of participants by group.

Variable	Tai Chi (*n* = 20)	Control (*n* = 19)
Age (yrs)	47.20 (6.99)	54.16 (6.09)^*∗*^

Gender, *n* (%)		
Female	17 (85.0%)	18 (94.7%)
Male	3 (15.0%)	1 (5.3%)

Height (cm)	160.81 (5.28)	158.14 (6.89)

Weight (kg)	59.41 (12.18)	59.34 (11.49)

BMI (kg/m^2^)	22.83 (3.60)	23.59 (3.42)

Education level, *n* (%)		
Middle school diploma	9 (45.0%)	8 (42.1%)
High school diploma	2 (10.0%)	0 (0.00%)
Junior college/associate bachelor	4 (20.0%)	4 (21.0%)
Bachelor's degree	4 (20.0%)	6 (31.6%)
Master's degree or higher	1 (5.0%)	1 (5.3%)

History of depression (yrs)	12.20 (7.49)	12.89 (6.20)

Use of medicine (yes), *n* (%)	16 (80.0%)	19 (100%)^*∗*^

Psychotherapies (yes), *n* (%)	8 (40.0%)	10 (52.6%)

*Note.* Data are reported as means (standard deviation) or percentage (%); *F* = female; *M* = male; MBI = body mass index. ^*∗*^Significant difference between groups (*p* < 0.05).

**Table 2:** Differences in executive functions and physical fitness between the Tai Chi group and the control group across times.

Variable	Tai Chi (*n* = 20)			Control (*n* = 19)		
	Pretest	Mid-test	Post-test	Pretest	Mid-test	Post-test
Stroop test						
Cong. (s)	22.10 (5.00)	21.45 (5.20)	18.75 (3.77)	22.58 (3.92)	22.89 (5.17)	21.68 (4.15)
InCon. (s)	38.35 (10.47)	37.75 (9.47)	30.65 (5.81)	39.63 (6.02)	38.95 (7.07)	38.21 (5.09)

TOL task						
TMS	29.85 (17.04)	17.75 (9.49)	13.10 (8.58)	33.58 (15.43)	29.05 (15.31)	27.47 (14.70)
TCS	3.85 (2.25)	5.95 (2.09)	6.65 (2.01)	4.00 (2.13)	4.89 (2.00)	4.79 (1.87)
TIT (s)	63.71 (51.48)	74.65 (65.27)	55.49 (75.09)	73.11 (53.25)	49.19 (35.44)	56.92 (49.00)
TET (s)	209.79 (74.35)	149.26 (51.60)	125.80 (48.27)	228.31 (78.83)	197.53 (55.72)	193.11 (57.37)
TPST (s)	273.50 (82.86)	223.91 (74.34)	181.29 (98.39)	301.43 (84.64)	246.72 (64.17)	250.03 (60.45)

WCST						
NCC	5.65 (1.18)	5.80 (0.89)	6.00 (0.00)	4.32 (2.24)	4.74 (2.18)	5.16 (1.61)
PR	13.30 (12.77)	7.20 (3.12)	6.00 (2.36)	17.53 (12.26)	13.95 (11.22)	12.26 (11.28)
CLR	68.70 (13.66)	69.45 (8.99)	67.80 (8.44)	60.00 (14.63)	60.84 (15.47)	66.84 (18.03)
FMS	0.60 (0.75)	1.05 (1.73)	0.50 (0.89)	0.84 (1.21)	1.00 (1.25)	1.26 (1.59)

CRF	24.65 (6.60)	27.43 (6.61)	30.15 (3.13)	23.92 (8.48)	22.36 (10.80)	23.86 (9.12)

MS (rep.)	6.61 (4.53)	9.61 (4.72)	11.83 (4.30)	7.17 (4.50)	7.56 (5.90)	8.06 (5.33)

ME (rep.)	5.00 (4.60)	7.33 (4.98)	10.22 (5.57)	6.11 (5.14)	6.61 (5.39)	7.06 (5.16)

Power (cm)	102.16 (17.32)	116.51 (18.70)	126.21 (17.11)	101.05 (25.49)	100.35 (30.07)	113.56 (26.86)

Flexibility (cm)	5.34 (7.61)	8.29 (8.22)	10.66 (7.84)	7.01 (11.17)	7.99 (10.36)	7.68 (11.68)

*Note.* Data are reported as means (SD); Cong. = congruent condition; InCon. = incongruent condition; TOL = Tower of London; TMS = total move score; TCS = total correct score; TIT = total initial time; TET = total executive time; TPST = total problem-solving time; WCST = Wisconsin Card Sorting Test; NCC = number of categories completed; PR = perseverative responses; CLR = conceptual-level responses; FMS = failure to maintain set; CRF = cardiovascular fitness; MS = muscular strength; rep. = repetitions; ME = muscular endurance.

## Data Availability

All of the data generated during the current study are available from the first corresponding author (F.F.R) upon reasonable request.
